# Identifying Positive Practices to Institutionalize Social Innovation in the Malawian Health System

**DOI:** 10.34172/ijhpm.8141

**Published:** 2024-12-09

**Authors:** Lindi van Niekerk, Lenore Manderson, Nedson G. Fosiko, Andrew Likaka, Carla P. Blauvelt, Barwani Msiska, Susan Rifkin

**Affiliations:** ^1^Department of Global Health and Development, London School of Hygiene and Tropical Medicine, London, UK.; ^2^Chembe Collaborative, Los Angeles, CA, USA.; ^3^School of Public Health, University of the Witwatersrand, Johannesburg, South Africa.; ^4^School of Social Sciences, Monash University, Melbourne, VIC, Australia.; ^5^The Malawi Ministry of Health, Lilongwe, Malawi.; ^6^VillageReach, Lilongwe, Malawi.; ^7^Malaria Alert Centre, Kamuzu University of Health Sciences, Blantyre, Malawi.

**Keywords:** Innovation, Scale, Health Systems Strengthening, Africa, Government, Human-Resource Management

## Abstract

**Background:**

Governments worldwide are increasingly interested in scaling up effective public health innovations, but it is not always easy to institutionalize innovations, arising outside the public health system, as a part of national delivery. Evidence on how country governments can practically achieve this is limited. This article describes the institutionalization of the Chipatala Cha Pa Foni (CCPF, Health Center by Phone) social innovation into the Malawian public health, and identifies positive institutional practices that local actors drew on to achieve this.

**Methods:**

A positive-oriented interdisciplinary multi-method qualitative case study design was adopted. Data were collected from key informant interviews, observations, and documents over 18 months. A composite social innovation framework, informed by institutional theory and positive organizational scholarship, guided the thematic content analysis.

**Results:**

Four clusters of positive institutional practices aided the institutionalization of the innovation: building high-quality relationships; creating opportunities for experiential interaction; cultivating hope; and logic attunement and awareness. We describe how these four practices operated together as a process of "everyday creativity" to achieve institutionalization. We illustrate the importance of high-quality relationships, marked by respect, mutuality, and appreciation, as the foundation upon which hope can be built and the creativity needed for institutionalization to flourish. National ownership and sustainability of innovations are enhanced when implementation and institutionalization approaches are attuned to the logics inherent in national identity.

**Conclusion:**

In this article, we highlight the importance of institutional and interpersonal dynamics in the institutionalization of social innovation in health systems.

## Background

**Key Messages**

**Implications for policy makers**

- Institutionalizing social innovations as part of the public health system in low- and middle-income countries (LMICs) is achievable. Although material resources are important, the value of human resources and positive institutional practices are critical.
- Institutionalization approached as a creative process provides new opportunities for collaboration, relationship building, and creativity across traditional hierarchical structures.
- Adopting a positive orientation or affirmative perspective in health systems practice supports the identification of otherwise hidden resources that can support innovation.
- Awareness of the operating and dominant logics implicit in the culture and context is key for mutually beneficial implementation and institutional partnerships between government and non-state actors.
- Implementers could adopt no-cost positive institutional practices when they lead or execute an institutionalization process.

**Implications for the public**

Social innovation offers an opportunity to strengthen low-resource health systems by leveraging the potential of citizens and governments alike. However, for social innovations in health to have a transformative and sustainable impact, adopting and institutionalizing them as part of the national health system is important. Very little empirical research exists to guide innovators and governments on how to achieve the adoption and institutionalization of social innovations. In this article, we present several positive institutional practices that can be used by governments and innovators to achieve this goal. These practices highlight that people can be a significant resource towards institutionalizing innovations, even amid material resource constraints.

Governments worldwide are increasingly interested in scaling up effective public health innovations to realize strong resilient national health systems and enhance population health impact. However, for many government actors and policy-makers in low- and middle-income countries (LMICs), translating innovation into policy is challenging, due to competing demands, resource constraints, and complicated bureaucratic processes. These factors can make public health systems resistant to innovations developed from outside the system.^1,2^

 Over the past decade, numerous organizations, universities, and agencies have identified and supported innovations, often developed by non-state actors, that have enhanced access to and quality of health services.^3-6^ Most commonly, non-state innovations have pursued scaling efforts via commercial pathways, at times at best establishing an affiliation with the government health system. However, the inherent transformative impact of these innovations cannot be achieved without embedding and integrating them within state institutions, including the public health system.^7^ The need for government adoption and institutionalization of non-state innovations has spurred investigations into pathways, practices, and approaches to assist in scaling innovations in the public sector, including the approval and accreditation of innovations and their procurement and purchasing through public-private partnerships.^3,8^ Empirical evidence on the scale-up of innovations by LMIC governments is limited and focuses on material realities such as resource availability.^1,9-12^ Practical management guidance for government actors wishing to integrate innovations within their public health systems is lacking.^5,13,14^

 One particular type of innovation, social innovation, has been described with promise and potential to support the achievement of Universal Health Coverage in LMIC health systems.^15-17^ Social innovation, as both process and outcome, is defined as an “agentic, relational, situated, and multi-level process to develop, promote and implement novel solutions to social problems.”^18^ It results in changes in basic routines, resource and authority flows, or beliefs of the system in which the innovation is embedded.^19^ Two paradigms or approaches to social innovation exist—the technocratic and the democratic. The technocratic paradigm emphasizes achieving greater service effectiveness or efficiency; the democratic paradigm goes beyond this to achieve transformative socio-institutional changes.^20^ Two theories closest to the democratic paradigm of social innovation, as identified in a scoping review,^15^ are neo-institutional theory and positive organizational scholarship. These theoretical streams contribute to understanding how social innovation can be institutionalized within a government health system.

 Chipatala Cha Pa Foni (CCPF, Chichewa), translated as Health Center by Phone, is a social innovation that was successfully institutionalized, fully adopted and delivered by the Malawi Ministry of Health (MoH) as a part of the national health system.^21^ The innovation was the brainchild of two Malawians who proposed addressing access challenges to primary care services by connecting nurses telephonically and via text messages to community members, so to provide Malawians with accurate and timely health information to better inform health-seeking behavior. The CCPF hotline, initiated before the COVID-19 pandemic, sharpened interest in communication technologies to meet health needs. CCPF continues to provide health information and referral guidance on all health topics across all 28 districts in the country. The hotline has had proven impact on sexual reproductive health, HIV, maternal health, child health, and nutrition indicators, eg, 52% of CCPF unmarried users used a condom versus 29% of non-users (*P* < .001).^22^ It played a key role in Malawi’s response during the COVID-19 pandemic.

**Figure 1:**
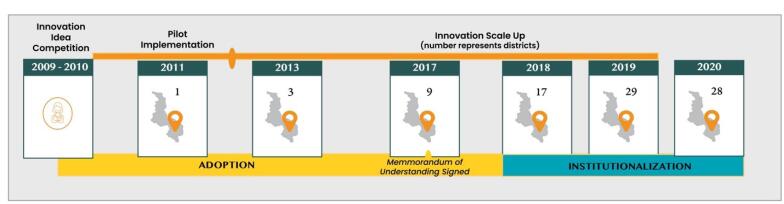
Chipatala Cha Pa Foni Over Time.

 CCPF was first implemented in 2011 by an international non-governmental organization (NGO), in the local district hospital in one district in Southern Malawi. Initially, the call center was run by health surveillance assistants (community health workers). CCPF’s reach grew steadily (Figure 1), and through support from other implementers in Malawi (eg, bilateral agencies), the innovation was extended to eight districts by 2016. In 2017, the Malawi MoH entered a Memorandum of Understanding with the implementing NGO, with an agreement that the Ministry would institutionalize CCPF as part of the public health system.^23^ This meant that the innovation was to be regarded as fully owned, paid for and operated by the government, and its coverage was extended to all Malawians nationwide.

 In support of institutionalization, the call center was moved from the original site in Southern Malawi to Lilongwe (the capital city), and located on the MoH premises, so demonstrating MoH’s ownership. To meet the requirements of the Malawi Medical Council, the call center was staffed with nurses and enabled by a technology platform that facilitated the provision of MoH-accredited health information. A government institutionalization lead was appointed, supported by a full-time seconded NGO representative. In addition, a cross-sectoral steering committee was established, to meet on a quarterly basis, and included all state and non-state partners involved in the social innovation. Full institutionalization of CCPF as a government-owned and led initiative had been effected by December 2020.^24^

 The purpose of the research described in this article was to study the institutionalization process of the CCPF within the Malawian public health system. We focus on the positive institutional practices that local actors drew on to steer institutionalization, which led to the acceptance and ownership of CCPF as integral to the public health system.

## Methods

 The study was positioned within the field of health policy and systems research. Instead of focusing on hardware or instrumental dimensions, it focused on the non-instrumental or software dimensions of health systems.^25^ The study was conducted using an interdisciplinary, positively oriented, qualitative case study design. Social innovation is an evolving process and highly context-bound, and the exploratory and explanatory potential of a case study meant it was particularly appropriate.^26^ We did not envision assessing causality via experimentation; rather, the working hypotheses were considered to be context and time-bound, shaped by multiple interacting factors, events, and processes. The study aimed not to establish generalizability. but to enable an understanding of the patterns and mechanisms of institutionalization within the context of Malawi’s decentralized health system.^27,28^ The case study provides clues to potential transferable processes in other similar health systems^29^ facing multiple resource constraints and geographic challenges that hinder people’s access to health services.

 An initial stakeholder mapping exercise identified all actors involved in the adoption and institutionalization process and clarified their different roles. Participant selection was defined by three categories: type (implementer, government actor, contributors, and observers); level (top-level, mid-level, and frontline), and extent of involvement (direct or indirect). Data were collected from all relevant actors, including the implementing NGO representatives, government representatives, project partner agencies, and others involved in CCPF. Additional participants were invited for interviews during the research. To control for positive bias, interviews with actors who may have conflicting, opposing, or negative views were sought out. In total, 68 interviews were conducted with 54 people over 18 months, so capturing progress in institutionalization. Twenty-nine actors were representatives of the Malawian MoH, working at district and national levels from all departments affected by the innovation. Interviews lasted 30–60 minutes and were conducted in person, in English; all except one were recorded and transcribed. Interview data were supplemented with observations and document reviews, including evaluation studies, monthly progress reports, presentations, promotional materials, and health policies. Observations focused on the day-to-day management processes, group meetings (steering committee meetings), actor roles, reactions, and contributions. Data collection ceased when saturation was reached, with no new information shared; all data were triangulated.

 To guide data analysis, a multi-level composite framework informed by theory from Institutional Work^30,31^ and Positive Organizational Scholarship^32,33^ was adapted.^18,24^ “Practices” are defined in Institutional Work as the “embodied, materially mediated arrays of human activity organized around shared practical understanding.”^34^ The synthesis of Institutional Work Theory and Positive Organizational Scholarship provides a positive orientation or affirmative bias toward the study of positive phenomena so that their characteristics, processes, and enablers can be better understood.^31,35^ The orientation does not ascribe to “reckless optimism” or “ignoring the negative”^36,37^; rather it focuses on institutional practices (human behaviors, routines, and patterns) that express mutually constitutive experience and social good.^31^ We illustrate this below.

 By using these theories, we were also able to account for institutionalization as occurring at three levels: micro-individual, meso-organizational, and macro-institutional. Data were collected on each level at each subsequent data collection round. Thematic analysis was conducted using deductive and inductive strategies. Five positive institutional practices used by actors to support institutionalization emerged inductively. Although data were collected in three cycles, we focus here on the meso-level without describing changes chronologically.

## Results

 The research identified four clusters of positive institutional practices utilized by the Malawi MoH, which supported the integration of the innovation. These were: building high-quality relationships; creating opportunities for experiential interaction; cultivating hope; and logic-attunement and awareness. We discuss these below in practical terms.

###  Building High-Quality Relationships

 CCPF is a multi-faceted technology-enabled social innovation, integrating ten technical areas of the MoH including health promotion, quality management, and clinical services. To achieve institutionalization, multiple cross-sectoral relationships were required, including support from different government departments, the community, and private for-profit and international non-state sector partners. While the breadth of relationship-building was significant for successful implementation and coordination, the depth and quality of the relationships played an important supporting role. According to interviewees and from observations, relational depth was achieved through three practices: respectful engagement, mutuality, and appreciative attention.

 The practice of respectful engagement^38^ and respectful behaviors in an organizational context is defined by how people affirm the worth, value, and dignity of others,^39^ and conveys acceptance and genuine interest; it has been linked to cultivating higher levels of creativity in individuals and teams.^40^ Participants considered respectful engagement to contribute significantly to the quality of the relationship between the NGO and the government actors, and to be important in gaining the government’s willingness to adopt and institutionalize the innovation. Government actors experienced ‘respectful engagement’ through the willingness by the NGO team to share information, effectively listen, and take their input on board to adapt the implementation for institutionalization:

 “*I don’t know how I can call it...the willingness of our colleagues from [NGO]. I think for me, right from the time we started with [country director], they were always sharing information. And I remember even there were times when they would want to not do it in the correct way and I would guide them and say no, this is the proper way. So, they were appreciative in saying thank you so much for guiding us to do the proper thing. So, this is how we were able to inform our other colleagues at the ministry so that it became embraced”* (Interviewee 010, Government).

 “*But fortunately, [country director] and the other one [monitoring officer], they are such good people. When the director introduced me to Chipatala Cha Pa Foni, we have been working very closely. My office is relatively busy, but they do understand me [laugh]. Their understanding is something else that I don’t take for granted. Some of the things we have to do may be dragging but they always come in to help move those things move on. And they can talk better about how we are trusting each other but I think I like the openness. When I ask questions, I always get answers. So that builds the trust and the understanding that this office is also busy”* (Interviewee 015, Government).

 Government health service actors at both central and district levels considered such respectful engagement unique, contrasting with their usual experience when dealing with non-Malawian agencies wanting to implement initiatives in their country. Government actors often had little opportunity to provide input in shaping programs, and even at the national level, they often perceive external organizations to impose innovations and the behavior of their staff to be imperious. This reduces government ownership of non-Malawian innovations or projects and government willingness to engage with them: *“We don’t like the ideas to be imposed. First of all, it should be that the both of us should understand and accept (the ideas)*” (Interviewee 014, Government). And again:

 “*A partner comes in and implements the projects themselves without the ministry or without the department working with them. The people whom they are working with on the ground, they always think, okay, we are doing this because the partner is seeing us. But when the partners leave, there’s nobody to supervise them and they don’t own it. It’s like the partner was the owner so when he goes everything goes”* (Interviewee 024, Government).

 In this study, the relationship between the NGO and government was characterized by *mutuality*, defined as “reciprocal transactions and exchange, mutual influence and responsiveness and a sense of common purpose.”^41^ These relationships embody Buber’s^42^ notion of “I-Thou,” in contrast to the “I-It” relationships found in the workplace, where the “other” is related to as something to fulfill an objective, not as a whole person.^43^ In institutionalizing CCPF, the NGO and government actors accepted mutual influence, equality, responsiveness, and dependence to achieve their shared goals, as particularly well represented in the relationship between the government lead and the NGO implementation lead. This mutuality fostered and heightened the positive emotions (see below) required for advancing the day-to-day work of institutionalization, even in the face of challenges and obstacles. Mutuality was a resource in the institutionalization process, generating a sense of shared identity in CCPF and allowing actors to feel they were in this process together:

 “*So, we are a team. Already we have a small team here, me and [technical advisor] and my director are always giving me the support I need. I like her when she challenges me to say, ‘you can do this,’ so that is motivating already”* (Interviewee 014, Government).

 Mutuality was expressed in terms of teamwork and shared leadership. Shared leadership deviates from the traditional hierarchical style of leadership in healthcare, where one person exerts downward influence. Sharing leads to more dynamic co-leading, with team members acknowledging each other’s contributions.^44^ In steering committee meetings, this was particularly evident. The government-appointed lead and meeting chair would regularly defer to others, giving them the opportunity to suggest ideas and take the lead on aspects of innovation institutionalization that fell within their remit or expertise. Two new government actors emerged who played key leadership roles. Both leveraged their deep understanding and prior experiences of institutionalization, and they were able to see clear opportunities to support institutionalization and future possibilities for synergy with other programs.

 The third practice that helped to build high-quality relationships was appreciative attention. “Appreciative,” as used in the tradition of appreciative inquiry,^45^ refers to seeing or noticing generative dimensions in organizational life – “things that give life (health, vitality, and excellence) to living systems” and valuing each individual’s gifts.^46,47^ Appreciative attention was displayed in several ways at quarterly steering committee meetings. These were all started by a meeting participant offering to say a prayer (in Chichewa or English), expressing gratitude to God for the day, thankfulness for the opportunity to be engaged, and praying for wisdom to know how to proceed. In a deeply Christian country, this was a welcomed act. Following this, the meeting host, either the government lead or a representative, would welcome each attendee by name and express gratitude for the time they had set aside to attend. Gratitude would further be expressed to each of the cross-sectoral non-governmental partners represented, emphasizing how CCPF would not be possible without their support. The various technical government departments were also acknowledged for their time investment and contribution, and the uniqueness of stakeholder/partner collaboration was highlighted. The government lead would subsequently recast the vision for CCPF and emphasize the novelty and opportunity for this innovation to make a tangible difference in the lives of millions of Malawians. In this way, he always identified CCPF as unique, and emphasized the privilege to be involved. At each meeting, positive news updates were shared prior to challenges: the ever-increasing number of Malawians benefiting from the innovation; recognitions received through external (national or international) awards; an invitation received to present at an international forum. These acts of affirmative attention created a general positivity among participants and likely contributed to their future creative behavior:

 “*The MoH is looking forward to taking it up. If a district has eight health facilities, we want them to consider CCPF to be the 9 th because Chipatala Cha Pa Foni is part and parcel of all the health facilities in Malawi. We just have to make it that way and it will require us to advocate. There will be some challenges but with all these ideas that I hear, I am very confident. When we were charged by the MoH, as the [name] Department to facilitate or promote CCPF, we were confident because we had people behind us. You know, when you have people behind you, even if you have all these challenges, I don’t expect to be disappointed because of you people. Your presence here gives us courage. So, with those few remarks, I just want to say thank you very much and I look forward to all the discussions. Your input is very valuable here” *(Government Chair 1, Meeting 29.06.2018).

 “*[1 year later] The collaboration partnership is really key, and it needs to be prioritized. It is important to communicate to the community that this is something unique, the first of its kind”* (Government Chair 1, Meeting 26.02.2019).

 “*[1 year later] The mandate we have been given is to make a project into a program. We are blessed by the senior management, which was endorsed by the same office, it has been successful as a project but now has to be taken as a program”* (Government Chair 2, Meeting 26.03.2019).

###  Creating Opportunities for Experiential Interaction

 Educating stakeholders by communicating information about a new innovation enables buy-in and support. To inform all stakeholders, the NGO-government team invested in communication materials (brochures, videos, and promotional t-shirts) and sent monthly monitoring data to all stakeholders, as well as conducted two impact evaluations reports. Uniquely, the education and communication work extended beyond information sharing to include an experiential dimension. Several government actors, involved from the beginning of CCPF, noted the impact of their visit to the pilot implementation district in a rural district in southern Malawi, as a key catalyst for government actors to achieve the contractual adoption of the innovation. Building on this initial encounter, ongoing opportunities were created for stakeholders to learn about the initiative through experience. Once the CCPF hotline center was moved to the capital Lilongwe, government representatives and project partners could visit the center and hear directly from nurses. Respected community leaders (chiefs, village headmen) were also invited to attend the steering committee meetings, bringing their own first-hand testimonies of the impact of CCPF on their communities. These encounters generated strong emotional commitment by stakeholders to ensure successful institutionalization:

 “*When it was in Balaka I had an opportunity to visit the district to appreciate what CCPF, the Chipatala Cha Pa Foni was about. And I had an opportunity to go there with the then Director of Planning. Because I said let’s go together so that you can appreciate what [the innovation] is doing. So, when we went there and we were able to listen to a phone call where a client was phoning and getting information and the Director of [name] was amused and said ‘I think this is the way to go, not only to give information on reproductive health to people but I think we can do it also with the other health areas’”* (Interviewee 010, Government).

 Ritualized social interaction at the quarterly steering committee meetings also allowed people to share experiences. These meetings were open to all cross-sectoral stakeholders, approximately 15–25 people. The meeting structure closely aligned with Furnari’s concept of “interstitial spaces of micro-interactions”: a catalyst (a facilitator, host, organizer), through continuity of presence, provides a structure and encouragement, so creating shared meaning and identity among actors.^48^ In meetings, this was core to the creativity involved in supporting institutionalization. The facilitated spaces provided a respectful but non-hierarchical environment in which stakeholders could think outside of institutionalized scripts and power dynamics, and actively address emerging challenges. This led to a wide variety of creative solutions, such as identifying alternative funding streams and leveraging timely political windows of opportunity. Although the strength of the ties between participants varied, new participants enhanced the cohesion and creativity of the group, stimulating different perspectives and ideas. By “facilitating a shared space for creativity,” new technical and financial resources and ideas emerged to address challenges in the existing resource-constrained health system. The shared space also supported generating ownership among government actors of CCPF as an MoH initiative.

 “*The values that I think played a role, the first one would be inclusiveness, to make sure that everyone is included and is participating in the establishment of Chipatala Cha Pa Foni. Whether you have got money, or you got resources, you need other people also to push you in another area or to cushion in other areas. So, partnership and collaboration are other values that I take to be very key, and respect is another thing. I don’t underrate people as far as I am concerned and I think when we meet as a group, we want to work in that fashion. When you come into that meeting, you will see that people that are mixing up there, are at different levels in terms of their hierarchies” *(Interviewee 015, Government).

 “*I don’t know where I was first involved but I found it already there. My colleague was the first one to be involved in one of the meetings and I also have been to attend one of them. Ja. It was quite good. And after that, it is like we are moving together, with the clinical department. Ja, so we share ideas and when there is a meeting we go and then see how we are moving forward”* (Interviewee 009, Government).

###  Cultivating Hope 

 Being positive and optimistic about innovations in the initial stages is much easier than over time, during a complex process of institutionalization. The CCPF innovation required change within the government’s institutional roles, routines, authority flows, identities, and meanings.^24^ This led to obstacles. CCPF lacked funding to absorb all 28 nurses as part of the government establishment, faced resistance from several technical departments to make changes to embed the innovation, and faced skepticism of the leadership approach. Several government and country actors, directly or indirectly involved, were skeptical, and acutely aware of the difficulties of institutionalizing and sustaining the innovation. There was no shortage of justifiable reasons to halt this undertaking on multiple occasions, especially as the completion deadline approached.

 The core government team however was hopeful that the institutionalization process would succeed, and participants expressed this at each steering committee meeting and interaction. “Hope,” as Fredrickson states, “creates the urge to draw on one’s own capabilities and inventiveness to turn things around.”^33^ Hope does not arise where circumstances are perceived as safe or easy, but rather, it comes alive in dire situations, in which people continue to yearn for better.^33^ It differs from optimism as it critically assesses the situation and finds new pathways forward. In this study, “cultivating hope” appeared as a practice, rather than an emotion, as an active choice made by government actors. The unrelenting hope of a small number of government actors, shared explicitly during steering committee meetings, had an amplifying effect on the broader group. This is consistent with Ludema,^49^ who described the act of hoping not as solitary, but rather as an inclusive act inextricably linked and essentially interdependent. That is, as people tap into life-giving relationships, they gain a sense of being carried and supported by others, and so become more generative and contribute to the generativity of others.^49^ Attendees of the steering committee would leave the meeting, after intense discussions of the challenges, with a heightened conviction that their actions and efforts could make institutionalization a success (see further below).

 “Gathering” per se would not be sufficient for hope to develop as a group-level resource. As described above, high-quality relationships and shared interactions and experiences created the fertile ground in which hope was nurtured and sustained. Through dialogue anchored in quality relationships, hope was cultivated, and it became possible to discover previously unrecognized and new possibilities.^49^

 “*I think when we get to have a strategy meeting and he says for instance: ‘[name], I am not worried about money. I know this thing has to run and it will run. Money will be made available, that is the least of my concerns.’ At one point I was concerned that the budget that was passed represents, maybe 10% or something of what it takes to run CCPF. But if he is quite confident to say that is not his problem, that is not his worry, then why should it give me a headache (laugh). If he is quite confident and I am now talking to someone who is taking it on, then there must be something he is banking on and so I really shouldn’t have a headache about this. So, when you have those discussions with Dr [name], you go into the room nervous but as you are leaving the room, you go, mmm, I think I need to rethink it all and see and get to the level of confidence that he is. Yeah”* (002 Interviewee, NGO).

 “*On a scale of 0 to 10, how far do you think with zero, not at all, and 10 is being completely institutionalized, where is CCPF along that line?”*

 “*I think, 95%, yes. Maybe 95% would be too generous. The major thing is the hotline, all the things have been done, except the human resources. The human resource has not been fully recruited. Once the government recruits the human resources for Chipatala Cha Pa Foni, that would be 100%. The only thing that is remaining is the recruitment of the human resource. But with the human resource being crucial it cannot be 1%”* (015 Interviewee, Government).

###  Logic-Attunement and Awareness

 A final set of practices emphasized logic attunement and awareness, including symbolic ownership and collectivism. This was revealed from areas of contention which occurred within the collaborative partnership, rather than from the successes, and affected the extent to which government ownership of the innovation was achieved. Government ownership was regarded by Malawian interviewees as a proxy indicator of the likelihood that the innovation would be sustained as an integral part of the Malawian health system.

 Logics have been described as supra-organizational principles and patterns.^50,51^ Logics are taken-for-granted ways of being and doing in operation within a country, which influence organizational reality. They are often unapparent to a non-national entering a country, yet they play a critical role in processes, procedures, and customs.

 In institutionalizing CCPF, two logics were identified—a national identity logic and a development logic. National identity logic was rooted in a post-colonial, post-dictatorial government legacy, one in which Malawians strive to have full power and ownership of their country. This logic was associated with universality (ie, “for all Malawians”), durability, sustainability, and respect for cultural values. This is despite Malawi’s continued dependence on external funders; 62% of total health expenditure comes from external development partners.^52^

 A national identity logic was often experienced in opposition to the development logic, which interviewees described as being time-bound, driven by efficiency, for selected population groups, and often imposed on Malawians, sidelining government processes and dismissing cultural acceptance. Projects were held to the donor logic of initiatives achieving results, while limited in their lifespan, unlikely to be sustained, and only benefiting a select region or group. Programs, in contrast, were considered to be government-owned and led initiatives:

 “*I have forgotten the name. But, in terms of their implementation strategy, they implement by themselves. They came and introduced the project – ‘we have this project’ – and then we went on to do the necessary. They did not involve the facility team members. And those projects, honestly, have not been successful. Because why? They lack ownership. After the projects have shifted out, nobody knew what they were doing and then they failed to sustain the project. So, I think that is one of the problems that make projects fail if they don’t involve the owners of the facility. They fail to sustain the project because there is no one to carry over when they have phased out”* (Interviewee 036, Government – District).

 The CCPF institutionalization partnership included the government, a non-Malawian NGO, and international partners (bilateral agencies). Within this context, the NGO and international partners ascribed more closely to the development logic, while the government and Malawian private sector companies ascribed to the national identity logic. This contrast in logics was evident in relation to the expected speed by which institutionalization was to occur and the timeline for completion; the visibility of government officials as leaders of the initiative; and the extent to which district-level government officials were engaged in the process.

 Practicing symbolic ownership was a key factor in achieving national ownership of CCPF. Operating under a national identity logic, government actors indirectly involved or observing the CCPF institutionalization felt that there was not sufficient public perception that the government had adopted the initiative and that CCPF was already owned by it. Operating under a development logic, the NGO regarded ownership to be more transactional, achieved through clearly defined actions and implemented according to an agreed timeline. Government actors stated that, even two years after the MoU was signed, the innovation was still seen as a “project” external to the health system, driven by outsiders (to the government) and lacking the symbolic value and identity required as a government “program”:

 “*Okay for me, firstly the way I was taught for every activity that is being done by a partner, still the lead person should be someone from the government. Until now, we are not sure under which sector does it fall. It is completely being done by [the NGO] and we have two months to go now. And the plan is that when it’s time to hand over, there will be a 2-week period where they will be shooing that person who I think is not enough”* (Interview 045, District Government).

 Practical ways in which symbolic ownership could be achieved were recommended by other Malawian nationals, including government actors and health implementers: ensuring a government-appointed person was the face of the initiative, especially when engaging with other levels of the health system; building flexibility within timelines such that they could be adjusted according to the realities and constraints of government; and adopting government processes and ways of doing (eg, timing meetings to the same frequency schedule as other government programs).

 A second practice influencing the approaches taken towards institutionalization, and their subsequent success, was awareness of the logic of personhood. Collectivism was presented as core aspect of Malawian personhood, as displayed in how Malawian actors were involved. Collectivism practiced and lived out by these actors transcended that of individual personal value or good strategic management practice. Rather, it was explained by the Malawian logic of moral personhood,*umbuntu*, in which “being a good person” cannot be achieved outside of a relationship with another, and required co-belonging and sharing.^53-55^ This contrasted with non-Malawian implementation and institutionalization approaches, founded on essentialist, individualist, and intellectualized ways of being a person.^56^ In this logic, the individual expert takes center stage, and achieving success and outcomes are idealized. These two logics influence how institutionalization is approached. A collectivist approach to institutionalization entails engaging all health system actors at different levels of the health systems. This extended beyond information sharing, to providing an opportunity for everyone’s input to be received and adjustments to be made to the innovation. The involvement of community traditional leaders and members of the district health management team were important in this respect. For Malawian actors, collectivist engagement was core to successful institutionalization and sustainability.

 “*Getting the buy-in from all the structures are critical before you actually bring in the program. And then introducing the tool or the approach and thinking about how to have it be implemented by the district health team. Then you get the buy-in. Most of the time it is us (external organizations) telling them (government) what we are bringing, and this is what it is going to do. It is ok to do that but it is also good to listen in terms because we have our rigid way of thinking as well, as in ‘this is the way it’s supposed to be done. When we encourage them to try it and fail and then correct the mistake together I think that is when you get the buy-in. Otherwise, it becomes an HP+ project, a Save project, a USAID project, and it is never their (government) project” *(Interviewee 054, Malawian health implementer).

 A more individualist-based approach would seek to involve people only if they could offer a direct contribution to institutionalization. The CCPF innovation was unique in that it directly linked beneficiaries/communities to the CCPF nurse-led hotline via mobile, thus negating the gatekeeping role of district health structures. Involving them in national scale-up was not considered essential time and expense, but as the institutionalization deadline was approaching, actors agreed that engaging the district health structures more meaningfully, in the context of a decentralized health system, could have provided many resources which the innovation was struggling to secure:

 “*We are using them as an office, but we are also trying to engage already existing structures in the district, so it goes back to what I was saying to say, ok because the office feels involved, the people feel they are involved and then they are more eager to say ok, I think we are in this together. Putting effort is much, much easier than thinking that you are operating in a parallel manner. We are doing our thing, and Chipatala Cha Pa Foni is doing its own thing, but that sort of engagement then removes the parallelism and puts you on the same track” *(Interviewee 053, District Government).

 “*The previous experience was important for us. The other thing which was also important was, this is the government, we shouldn’t say we are transitioning on this date. We shouldn’t dictate things. We should wait on them. If we don’t have money to run beyond the dates, we should just be honest with ourselves. Ok, we will just stop a little bit here and wait for you, but this is how Chipatala Cha Pa Foni works and it is important that just give them the value on their system. So, maybe they would know what department would be relevant. Not just now, but even for the future, you know. For me, this is prevention, and it is education. So, Health Education and Community Health, those were the very best departments who we were supposed to align with” *(Interviewee 007, NGO).

## Discussion

 This study adopted a positive orientation or affirmative bias, consistent with the theory of positive organizational scholarship and positive institutional work, to identify positive institutional practices used by actors to support the institutionalization of social innovation. Although this orientation may pose limitations to the research, we do not believe that it is any more limiting than the traditional negative, deficit, or constraint-focused bias applied in health systems scholarship. Rather, as highlighted above, a positive orientation supported the identification of useful and practical institutional practices that may otherwise have gone unnoticed.

 In general, practices are most powerful when they simply appear as a natural expression of who people are, what they do, and their ways of being and relating. Yet, as described by institutional theorists, these practices play an important role in providing insight into the “internal life of processes” and their influence on institutional change or resistance. In the scholarly literature, positive institutional practices are associated with enhancing the effectiveness of organizations and improving financial performance, quality of care, and client satisfaction.^57^ Positive institutional practices amplify what is good and buffer against challenges or deficiencies, enabling an optimal outcome.^57^ In this case study, although the innovation took place within one of the most resource-constrained health systems worldwide, actors were able to use positive institutional practices as intangible, social, and human-based resources to support successful institutionalization. Focusing on inherent positive institutional practices employed by health system actors emphasizes the value these actors offer health systems, beyond mere technical work and outputs.

 In previous health system institutionalization inquiries, the focus has been predominantly on describing the tangible, concrete, or hardware factors needed for this process eg, finances and financial mechanisms, staff, standard operating procedures, and technology. Existing toolkits guiding government actors wishing to institutionalize an innovation within the public health system have referred to “collaboration,” “partnership,” “gaining commitment,” and “ensuring ownership,” but it is rarely clear how this can be achieved. This study contributes to the literature on health system software (intangible) factors that influence institutionalization and provides a practical and no-cost way to support health system software dimensions, by focusing on intangible positive institutional practices.

**Figure 2:**
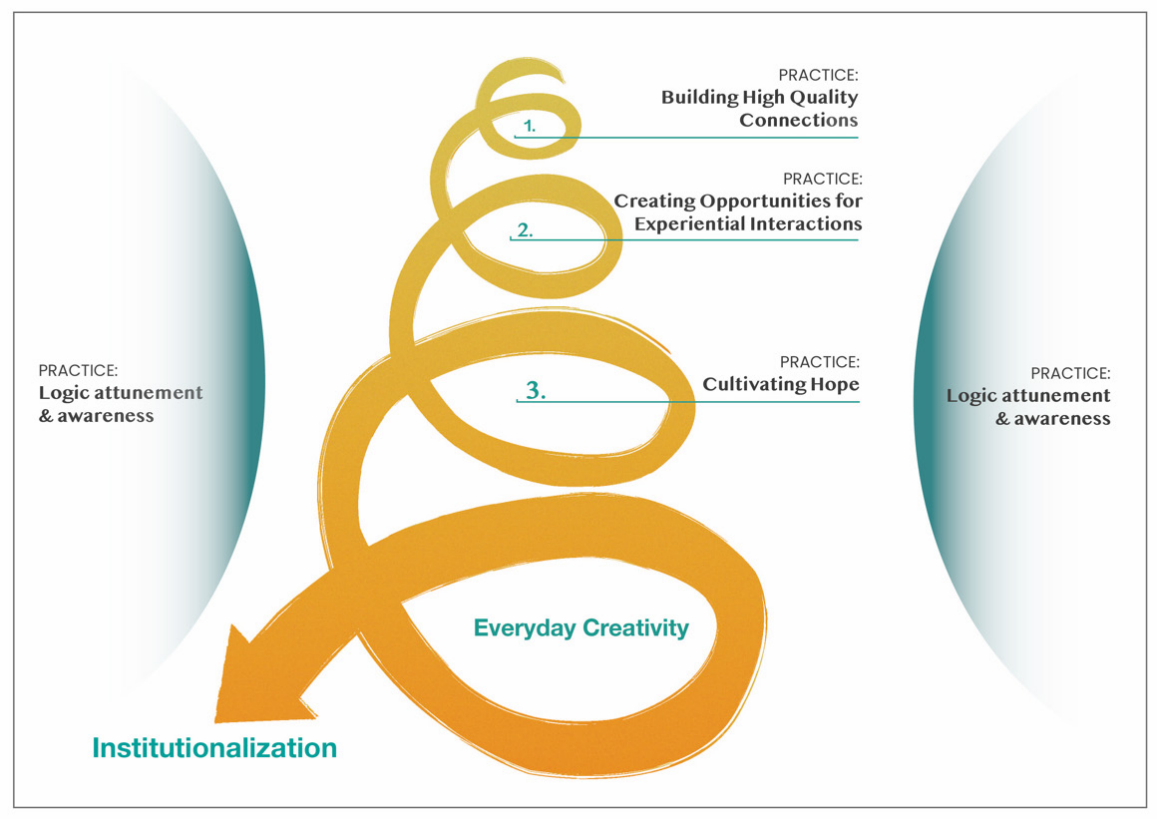
The Process and Practices of Institutionalization.

 The positive institutional practices presented in Figure 2 provide a road map to enhance institutionalization. In the Malawi example, the institutionalization process resulted in an ongoing need for actors to interact, brainstorm, develop solutions, and work around bureaucratic constraints to enable CCPF to be embedded within the government. Institutionalization is often perceived as bureaucratic, but in this case, institutionalization took on the style of the “everyday nature” of creativity and innovation, as diverse actors tested out multiple small ideas, acts, strategies, and reconfigurations to identify ways to institutionalize CCPF.^58^

 High-quality relationships, marked by respect, mutuality, and appreciation, are the foundation upon which the creativity required for institutionalization can flourish. High-quality relationships have a higher degree of emotional carrying capacity or resilience, ie, the ability to withstand tension or conflict, and a higher degree of openness to new ideas, influences, and divergent thinking.^59^ The institutionalization process disrupted entrenched actor roles formed by disciplinary or hierarchical lines, and created a more power-neutral space for ongoing social interaction and dialogue. This interaction became the foundation upon which hope could be cultivated. Hope, an overlooked health system resource, enabled the creative generation of ideas, strategies, and solutions, even when institutionalization seemed difficult or perhaps impossible.^60^ Hope enhanced the capacity of government and other actors to embrace the risks associated with innovation and so unlocked their agency to act. We expect that this enhanced capacity will make the future institutionalization of other non-state social innovations easier and more acceptable.

 The country context within which an innovation is institutionalized also needs to be recognized for more than its political, economic and social structures. It requires a sensitivity to national identity and personhood logics influencing actors and systems. Practical ways in which a greater awareness of personhood can be achieved is by the awareness of implementing partners of their own views and values. Ways of being are taken for granted, but through thoughtful self-reflection, awareness is attainable. In addition, non-national implementers need to invest more time to learn about the culture in which they find themselves and to question their own paradigms. Respect needs to be shown by following established country processes of engagement or acknowledging local leadership, rather than circumventing these for the sake of efficiency. By tailoring implementation and institutionalization approaches to be respectful and attuned to this, national ownership, and the uptake and sustainability of innovations, would likely be enhanced. Embedding a social innovation in the health system pushes the institutional boundaries of the broader system while enabling human-based resources to maintain the integrity of the system.

###  Limitations

 This study was conducted for doctoral research and was initially conceived as a comparative case study between two social innovations in different low-income country African settings. For practical reasons, a single innovation in one country was studied. All data were collected and analyzed by a single South African researcher, and this posed challenges for the validation of the findings. To address this limitation, the findings were discussed in an ongoing manner with Malawian researchers, and triangulation of data sources and methods was conducted. Findings from the two initial rounds of data collection were tested with respondents during the third and final rounds to ensure accuracy of interpretation. Analysis by induction is limited, and to validate these findings, testing these practices in other settings and projects would be beneficial. It would also be important to explore the interrelationship between the practices in more detail in future studies.

## Conclusion

 Institutionalizing social innovation in health systems in LMICs is achievable and could support health systems in achieving their goals to extend access to health services. The role and importance of positive institutional practices offers resource-efficient ways for institutionalization to be achieved. Local actors, drawing on a range of personal and relational behaviors, routines, and patterns, cultivated the intangible resources required for the successful institutionalization of the CCPF social innovation. Further research would be valuable to assess if these practices contribute to the institutionalization of social innovations in health systems in other settings.

## Acknowledgements

 We thank the following people for their contribution and support in this research study: from the Malawi MoH: Dr. Fanny Kachale, Dr. Rabson Kachala, and Mr. Isaac Dambula; from Kamuzu University of Health Sciences: Prof. Don Mathanga and Dr. Vincent Jumbe; from VillageReach Malawi: Upile Kachala, Lucky Gondwe, and Dr. Alinafe Kasiya; from the London School of Hygiene and Tropical Medicine: Prof. Lucy Gilson and Prof. Dina Balabanova; and for visual illustration: Claudi van Niekerk.

## Ethical issues

 This study received ethical clearance from the Ethics Review Board at the London School of Hygiene and Tropical Medicine (15476) and the National Council for Science and Technology in Malawi (NO.P.11/17/230).

## Conflicts of interest

 Authors declare that they have no conflicts of interest.
